# Supplementary material for: A four-season study quantifying the weekly external training loads during different between match microcycle lengths in professional rugby league
Source: PLoS One. 2022 Jan 31;17(1):e0263093. doi: 10.1371/journal.pone.0263093 (PMC8803197; doi:10.1371/journal.pone.0263093)
Supplement: Attachment — Submitted filename: Response to reviewers.docx [file pone.0263093.s002.docx]

Reviewer #1: Abstract
No indication about the type of external load parameters/instruments can be known by reading the abstract section. Therefore, it could be provided brief notes about this fundamental information to make more readable this independent section.

Thank you for your comment, the external load variables used within the study have been included in the abstract which now reads

“Commonly used external training load variables including total-, high-speed- (5-7 m∙s-1), and sprint-distance (> 7 m∙s-1) alongside combined high acceleration and deceleration distance (> 2 m∙s-2). Which were also expressed relative to time were collected using microtechnology within a repeated measures design from 54 male rugby league players from one Super League team over four seasons. 4337 individual observations across ninety-one separate microcycles and six individual microcycle lengths (5 to 10 day) were included.”

Introduction
Lines 58-62. Actually, the sole use of internal training load could determine limits as well as the sole use external loads. Therefore, for a complete training monitoring, both training loads should be considered. For this point, the following and recent study should be considered in the Introduction (and Discussion) section. Lupo C., Ungureanu A.N., Boccia G., Licciardi A., Rainoldi A., Brustio P.R. (2021). Internal training load monitoring, notational and time motion analyses, psychometric status, and neuromuscular responses in elite rugby union. International Journal of Sports Physiology and Performance. 5;16(3):421-428.

Thank you for the comment. We agree that for a holistic view of training load, a combination of both internal and external load measurements is important. However, we feel that this statement does not quite fit in the introduction whereby we state that previous studies assessing microcycle length have been limited by the sole use of internal training load as this does not provide an understanding of the training prescribed during different length microcycles i.e., components of the external load (Impellizierri, Marcora and Coutts, 2019).

Discussion
Lines 212-218. Limitations. The missing consideration of at least one internal training load parameters should be considered as a limitation. The grade of divergences between training leaded by different head coaches as well as practiced by different players would be better controlled by means of internal training load parameters such as RPE and Well-being perception (Lupo et al., 2021).

Thank you for the comment, we agree that the inclusion of internal load variables may provide a more holistic view of training load thus has been listed as a limitation of the study.

The section now reads “A second limitation of the study is the sole use of external load measurements, the inclusion of an internal load variable would provide a more holistic view of training load and variances between players and head coaches (Lupo et al., 2021)”

Practical application
Even though these results have been originated from a single team collection data (as fairly reported among limitations), more general practical applications could be provide to make the article more useful for physical trainers and coaches.

Thank you for the comment. We have added in practical applications for the microcycle comparison data but have stressed that the data may only be specific to the players and coaches of one club.

Reviewer #2: General comments

The paper aimed to quantify and compare commonly used external training load metrics between different between-match microcycle lengths; and to determine total weekly training load variation due to microcycle, individual and head coach.
The paper is generally well written based on sound literature, the methods are clear, detailed and replicable, the results well-presented and discussed with respect to the literature.
When using abbreviations, make sure you explain it first and then use the correct abbreviation throughout the paper.
Introduction
The authors kept in mind that this section is a development of the hypotheses of the study leading to the purpose of the investigation. The most relevant literature has been included, and I think the section flows with no major concerns.
Materials and Methods
Overall, methods and procedures are clear, detailed and replicable.
Results and discussion
I think the results and discussion sections of the study are well presented and discussed with respect to the current literature.
Figure and tables
Figures and tables should be clear and stand on their own. Make sure you check this.

Thank you for the comments they are greatly appreciated. We have ensured that all abbreviations have been explained previously in the paper namely “HDOP” in the methods section has now been explained as horizontal dilution of precision. We have also increased the resolution of figure 1 so now feel the figures and tables do stand on their own and the titles provide an adequate description to allow understanding.
